# Internal Jugular Vein Catheterization: The Landmark Technique versus Ultrasonography Guidance in Cardiac Surgery

**DOI:** 10.7759/cureus.4026

**Published:** 2019-02-07

**Authors:** Selcuk Kayir, Sertan Ozyalcin, Guvenc Dogan, Adem Ilkay Diken, Ufuk Turkmen

**Affiliations:** 1 Anesthesiology, Hitit University Erol Olcok Training and Research Hospital, Corum, TUR; 2 Cardiovascular Surgery, Hitit University Erol Olcok Training and Research Hospital, Corum, TUR

**Keywords:** cardiac surgery, landmark technique, ultrasonography, central venous catheterization

## Abstract

Background

Central venous catheterization is an invasive procedure that must be performed during cardiovascular surgery. The addition of ultrasound guidance to the catheterization technique has shown effectiveness in reducing complications because it allows for the visualization of anatomical variations prior to intervention and the continual visualization of the needle during the placement. The purpose of this study was to evaluate the effectiveness of needle-guiding ultrasound for internal jugular venous cannulation.

Method

Patients undergoing coronary bypass surgery at Hitit University, department of cardiovascular surgery, from January 2014 to June 2018, were included in the study. The patients were divided into two groups: those with catheterization with ultrasound guidance (Group U) and those with catheterization performed with the anatomic landmark technique (Group L).

Results

A total of 584 cases were investigated. The success of the procedure and complication rates for both methods were compared. Central vein catheterization with ultrasonography produced success and complication rates significantly better than those for catheterization using the landmark technique (p=0.04 and p=0.00001, respectively).

Conclusion

This study demonstrated that the use of ultrasonography for internal jugular vein catheterization for patients undergoing coronary bypass surgery significantly reduced the complication rates as compared to those of patients where the landmark technique was used for catheterization.

## Introduction

Central venous catheterization (CVC) is an invasive procedure that must be performed during cardiovascular surgery (CVS). Among the indications are hemodynamic monitoring, central venous pressure monitoring, blood transfusion and fluid replacement, a variety of drug infusions, hemodialysis, and hyperalimentation. There may be differences between operators and centers in the routes chosen for CVC. The most commonly chosen intervention route in CVS operations is the internal jugular vein (IJV). The subclavian vein and femoral vein are other anatomic regions where CVC can be performed [1-2]. Complications that may be observed during CVC procedures range from simple local hematomas to complications with mortal progression, including chylothorax, pneumothorax, hemothorax, or mediastinitis.

Until recently, CVC was performed using anatomical indicators (the landmark technique). Although the landmark technique is very standardized, even in the best hands, it has complication rates that cannot be reduced [3]. Currently, this technique is being replaced by methods using imaging modalities as an aid.

CVC guided by ultrasonography (USG) was first described by Legler [4-7]. This method ensures the visualization of anatomic variations before interventions, independent of location and continuous observation of the needle during cannulation. Thus, there are fewer needle passes, shorter catheterization times, and fewer complications. Venous interventions completed with USG are reported to have low complication rates [8].

The aim of this study was to compare the landmark technique versus USG for CVC in patients undergoing CVS in terms of successful intervention numbers, complications developed during and after the procedure, and the mean duration of the procedure.

## Materials and methods

Following approval from the Hitit University clinical research ethics committee, results from patients undergoing coronary bypass surgery in the department of cardiovascular surgery, from January 2014 to June 2018, were retrospectively examined. A total of 584 patients aged 18–80 years, in the American Society of Anesthesiologists (ASA) I–III, with platelet counts >50,000 mm^3^ and international normalized ratio (INR) <1.5 were included in the study. Patients with CVC in the preoperative period for any of a variety of indications, with platelet counts <50,000 mm^3^, INR >1.5, infection at the intervention site, valve or aorta surgery (as these patients had different levels of antiaggregant and anticoagulant drug protocols administered in the preoperative period), and planned carotid artery surgery were excluded from the study. The anesthesia forms, surgery notes, intensive care monitoring forms, and hospital database were used to obtain patient data.

Patients in the study were divided into two groups: those with catheterization with USG (Group U) and those with catheterization performed with the anatomic landmark technique (Group L). After patients were taken into the operating room for surgery, 5-derivation electrocardiography, pulse oximetry, and arterial pressure monitoring were performed. Patients had anesthesia induction and muscle relaxation, and after intubation, were placed in a slight Trendelenburg position while supine. The head was turned to the side opposite the CVC side. In both groups, local field cleaning was performed with povidone iodine. In Group U, a gel was spread on the USG probe, it was covered with a sterile sheath, and entry was beside the sternocleidomastoid muscle. In Group L, the landmark technique was used to insert the catheter.

The demographic data of the cases were examined. Preoperative hemorrhage profiles, platelet counts, and antiaggregant and anticoagulant intakes were compared. Additionally, the successful venous entry numbers, complications developed during the procedures, and technical successes were evaluated. An unsuccessful procedure was defined as catheterization not ensured after the puncture. Complications developed during the procedure were identified as palpable hematoma after the procedure and operation, carotid artery puncture, pneumothorax, or catheter malposition seen on radiological images obtained during or after the operation. Late period complications were determined from the clinical follow-up and file data of patients.

Statistical analysis

Statistical analysis was performed using the SPSS for Windows version 15.0 software (SPSS Inc., Chicago, IL, USA). Descriptive data were expressed in mean ± standard deviation (SD) and number and frequency (%). The student’s t-test was used for a comparison of quantitative variants. Qualitative variants were compared using chi-square tests or the Fisher’s exact test, as appropriate. The Pearson correlation analysis was used to examine the relationships between the parameters that conform to the normal distribution. A p-value of <0,05 was considered statistically significant.

## Results

This study investigated data from a total of 584 cases. The mean age of the subjects was 69.2±10.7 years. There was no statistically significant difference observed between the two study groups in terms of age or gender (p=0.3 and p=0.8, respectively). There was no significant difference between the two groups in the duration of the bypass operations (p=0.7). In Group U, 98.8% of patients had successful IJV catheterization. For these patients, 94.7% had right IJV catheterization and 4.1% had left IJV catheterization. Among the 1.2% of patients with unsuccessful procedures, right or left femoral vein catherization had been performed.

In Group L, 91.5% of patients had successful IJV catheterization. Of these patients, 85.2% had right IJV catheterization and 6.3% had left IJV catheterization. For the 9.5% of patients with unsuccessful procedures, right or left femoral vein catheterization had been performed. A comparison of procedure success between the two groups showed that CVC with USG gave a statistically higher rate of success (p=0.04).

There was no statistically significant difference between the two study groups in platelet counts, INR values, and antiaggregant and anticoagulant drug use in the preoperative period. The activated clotting time (ACT) values measured during cardiopulmonary bypass surgery were not identified to be significantly different in the groups (p=0.4) (Table 1).

**Table 1:** Characteristics of patients NS: Non-significant, ACT: Activated clotting time, INR: International normalized ratio *: p<0.05

	Group U (n=382)	Group L (n=202)	p value
Age (years)	68.7±12.5	71.2±9.8	NS
Gender (male)	51.8%	52.9%	NS
Duration of operation (minutes)	186.5±42.7	198.2±37.5	NS
Right internal jugular vein catherization (%)	94.7%	85.2%	NS
Left internal jugular vein catherization (%)	4.1%	6.3%	NS
Number of successful procedures (%)	98.8%	91.5%	0.04*
Preoperative platelet level	152.360±42.440	168.430±41.860	NS
ACT value during cardiopulmonary bypass	657±43	598±68	NS
Preoperative INR	1.12±0.3	1.08±0.4	NS
Preoperative antiaggregant use (%)	90.5%	89.7%	NS
Preoperative anticoagulant use (%)	99.1%	97.7%	NS

When complications after the procedure are investigated, eight patients in Group U and 18 patients in Group L were observed to have a palpable hematoma. Of those developing a hematoma during postoperative monitoring, 12 patients in Group L and eight patients in Group U resolved on their own with no late complications developing. There was a statistically significant difference observed in terms of hematoma development in Group L (p=0.03). One patient underwent jugular vein exploration due to hematoma growth in the postoperative period with vein repair procedure completed.

When groups are assessed in terms of carotid artery puncture, two patients in Group U and 15 patients in Group L had a carotid artery puncture identified. There was a statistically significant difference identified between the two groups (p=0.01). All patients with a carotid artery puncture were observed not to develop late complications.

With radiological imaging performed during the postoperative monitoring of patients, one patient in Group U was observed to develop pneumothorax of 10% or less with no hemothorax observed. In Group L, 10 patients were observed to have 10% or less pneumothorax. There was a statistically significant difference in terms of pneumothorax development. All patients with pneumothorax were monitored. All complications were observed to resolve with conventional treatment without developing late complications (Table 2).

**Table 2:** Complications rate NS: Non-significant *: p <0.05

	Group U (n=382)	Group L (n=202)	p-value
Palpable hematoma (n)	8	18	0.03*
Carotid artery puncture (n)	2	15	0.01*
Pneumothorax (n)	0	10	0,01*
Catheter malposition (n)	15	13	NS
Hemorrhage requiring surgical intervention (n)	0	3	0.05*

## Discussion

The total complication rate was found to be 6% in Group U while it was 24% in Group L. USG guidance revealed an 18% decrease in the overall complication rate. When the total complication rate was compared, USG guidance was more successful in the Landmark technique and statistically significant (p=0.0001). In the literature, central vein catheterization was shown to have mechanical complication rates of 5%-19%, thrombotic complication rates of 2%-26%, and infection complication rates of 5%-26% [9-11]. Many factors play a role in the development of these complications. Leading factors that affect the success of the procedure are morbid obesity, advanced cachexia, scarring developing at the intervention site linked to previous surgery or radiotherapy, comorbid diseases, and the experience of the clinician performing the procedure [12-15].

Ultrasound use during central vein catheterization provides advanced degree benefits to the clinician performing the procedure for the identification and assessment of the target vein in addition to an observation of the anatomic structures around the vein. This method was shown to increase the procedure's success in intensive care patients and severely reduce the procedure's complications in a variety of publications in the literature [16-17]. The main outcome of this study is that for heart surgery patients with very frequent use of antiaggregant and anticoagulants before and during surgery, USG use during preoperative internal jugular vein catheterization may prevent vital complications.

In our study in patients undergoing coronary bypass surgery, the success of jugular vein catheterization using the landmark method before the operation was identified as 91.5%. In studies, the success of this method varies from 85% to 99% [17-19]. In our study, carotid artery puncture with this method was identified as 7%; in the literature, this rate was identified from 3%-6%, which appears to be nearly the same as our study data. Additionally, pneumothorax and hemothorax were identified as 5% and 3% in our study, which appear to comply with values from other studies [19]. This retrospective analysis observed that the total complication rates fell from 26% to 6% when USG guidance is used instead of the landmark technique for catheterization with direct visualization of the IJV.

In our study, central venous catheterization accompanied by ultrasound was observed to have mechanical complications close to zero. In two patients, carotid artery puncture was identified. In our hospital, there is no contraindication in the preoperative period for cardiovascular surgery, with all patients undergoing coronary artery bypass surgery using daily 100 mg acetylsalicylic acid, 75 mg clopidogrel, and 6000 IU enoxaparin treatment preoperatively. After central venous catheterization with ultrasound guidance, eight patients were observed to have a palpable hematoma, with all hematomas resolving during monitoring. The cause of hematoma in these patients is considered to be linked to the use of antiaggregant and anticoagulant medication preoperatively. Pneumothorax was not observed.

Malposition is another complication that may be observed during central catheterization. In our study, all patients were assessed in terms of catheter malposition with 15 patients in the USG-guided group and 13 patients in the landmark method group observed to have catheters inserted into the jugular vein located in the opposite subclavian vein or rotating in the vein, with a location outside the right atrium. For all catheters with malposition, no function disruption was observed. From this aspect, the methods were not superior to each other in terms of developing malposition.

Studies in the literature with central vein catheterization using ultrasound guidance and the landmark method compared inexperienced clinicians. All clinician groups using USG were shown to have reduced complication rates independent of experience [19-21].

Complications linked to many causes, such as jugular vein path anomalies, the presence of collapsed veins linked to hypovolemia, and difficulty with jugular vein puncture due to carotid beats in patients with uncontrolled hypertension, may be eliminated with USG. In this way, the need for personal experience, which is at the forefront of the landmark method is reduced by direct visualization. Thus, during the training process, it is possible administrators may perform more reliable central vein catheterization.

In our clinic, standard catheters and unmodified ultrasound devices are used for central vein catheterization. The most important problem for these methods is the cost of obtaining USG devices. However, some studies have proven that in spite of these initial costs, central vein catheterization with USG is more cost-effective [22-23]. From this aspect, when advantages such as lower complication rates, requiring less personal experience, and higher procedure success are considered, it is clear that planning to obtain a USG device for central vein catheterization will be more cost-effective in the long term, especially for newly founded centers.

## Conclusions

As a result, central cannulation is a surgical procedure that must be performed in cardiac surgery. In our study, it was proven that the use of USG for internal jugular vein catheterization of patients receiving intense anticoagulant and antiaggregant treatment and hospitalized for coronary bypass surgery reduced the complication rates by a serious proportion. We suggest that ultrasound-guided central venous catheterization is a safe and effective alternative to the landmark technique.
